# Anomalous temperature dependence of elastic limit in metallic glasses

**DOI:** 10.1038/s41467-023-44048-7

**Published:** 2024-01-02

**Authors:** Yifan Wang, Jing Liu, Jian-Zhong Jiang, Wei Cai

**Affiliations:** 1 Department of Mechanical Engineering, Stanford University, Stanford, CA USA; 2 International Center for New-Structured Materials (ICNSM), and School of Materials Science and Engineering, Zhejiang University, Hangzhou, Zhejiang PR China; 3 School of Materials Science and Engineering, Fuyao University of Science and Technology, Fuzhou, Fujian PR China

**Keywords:** Glasses, Atomistic models, Metals and alloys, Mechanical engineering

## Abstract

Understanding the atomistic mechanisms of inelastic deformation in metallic glasses (MGs) remains challenging due to their amorphous structure, where local carriers of plasticity cannot be easily defined. Using molecular dynamics (MD) simulations, we analyzed the onset of inelastic deformation in CuZr MGs, specifically the temperature dependence of the elastic limit, in terms of localized shear transformation (ST) events. We find that although the ST events initiate at lower strain with increasing temperature, the elastic limit increases with temperature in certain temperature ranges. We explain this anomalous behavior through the framework of an energy-strain landscape (ESL) constructed from high-throughput strain-dependent energy barrier calculations for the ST events identified in the MD simulations. The ESL reveals that the anomalous behavior is caused by the transition of ST events from irreversible to reversible with increasing temperature. An analytical formulation is developed to predict this transition and the temperature dependence of the elastic limit.

## Abstract (web summary)

It is still challenging to study the atomistic mechanism of inelastic deformation in metallic glasses owing to their amorphous structure. Here, the authors report an anomalous temperature dependence of the onset of plasticity in metallic glasses at low temperature.

## Introduction

**Fig. 1:**
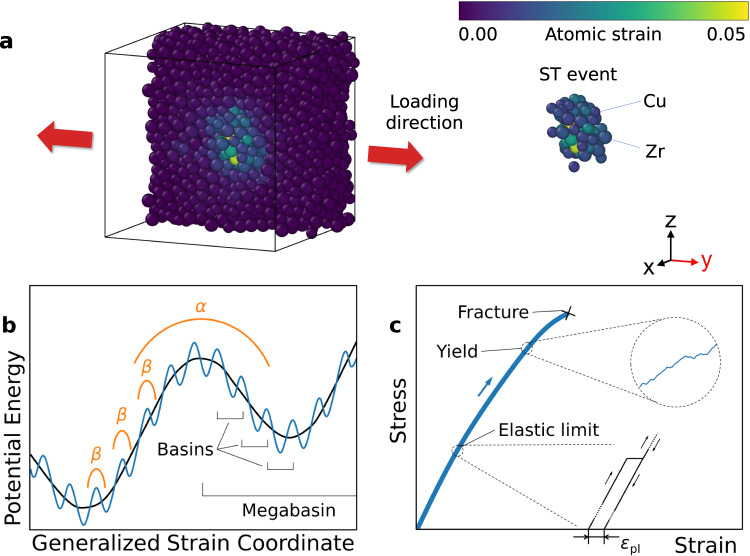
Macroscopic and microscopic mechanisms of metallic glass (MG) deformation. **a** A Cu_64.5_Zr_35.5_ MG configuration (Cu - small atoms; Zr - large atoms) of 5000 atoms. The red arrows indicate the uniaxial loading direction along the *y*-axis. Atoms are colored according to their von Mises atomic strain magnitude compared to the initial configuration before loading using the OVITO software^44^. Half of the atoms in the front are removed in order to reveal the shear transformation (ST) event at the center of the simulation box. **b** A schematic 1D potential energy landscape (PEL) showing *α* transitions between megabasins and *β* transitions between smaller basins. **c** Schematic stress-strain relationship of a typical MG tensile test, showing an irreversible ST event at the elastic limit and coalescence of ST events at the macroscopic yield strength (in the circle). The data in the circle is from the MD simulation of tensile test performed at 20 K. Arrows indicate the loading or unloading direction.

As an emerging engineering material, metallic glasses (MGs) exhibit attractive mechanical properties, especially a higher yield strength than their crystalline counterparts^1–4^, but the lack of ductility after yielding in tension limits their applications as structural materials^3^. Despite the significant efforts over the past decades^5–8^, the atomistic mechanisms of plasticity in MGs remain poorly understood^9,10^. The elementary step of inelastic deformation in MGs is thought to occur through structural rearrangements in a cluster of atoms (see Fig. 1a), named shear-transformation (ST) events^11–13^. However, it is still controversial whether or not the ST events can be attributed to local structural defects with well-defined size and geometrical features^14–16^. Large-scale yielding in MGs occurs through the formation of shear bands, believed to result from the interaction and coalescence of many ST events^17,18^. Given the existing controversies regarding ST events, a pre-requisite for understanding yielding in MGs is first to establish a concrete understanding of the incipient micro-plasticity^19^, where individual, isolated ST events govern the elastic limit (${\varepsilon }_{\lim }$), the minimal strain that produces detectable deformation irrecoverable by unloading, as sketched in Fig. 1c.

The potential energy landscape (PEL) provides a much-needed theoretical framework^20,21^ for discussing the dynamics of ST events in glassy materials. In particular, the PEL is envisioned to consist of large megabasins containing smaller basins inside (see Fig. 1b)^10,17^. The transitions between basins (i.e., metastable states) correspond to microscopic ST events (*β* relaxations), while the transitions between megabasins correspond to macroscopic plastic flow (*α* relaxations) through the accumulation of ST events^22^. The high-dimensional PEL (3*N*-dimension for an *N*-atom system) is often sketched schematically as an one-dimensional (1D) curve (e.g., Fig. 1b), which has several deficiencies for describing MG behaviors under the coupling of thermal activation and external loading. First, the 1D picture presumes that the system always goes through the same sequence of basins during loading and unloading, which can only be justified at the zero-temperature limit^23^. At finite temperatures, the system can jump to different neighboring basins and can take different trajectories in the (3*N*-dimensional) configuration space, according to the strain and temperature-dependent transition probabilities. Second, the 1D picture cannot quantitatively describe the change (e.g., ‘tilting’) of PEL^21^ by the applied external loading, which holds the key to predicting the rate of thermally activated ST events during deformation.

Here we use molecular dynamics (MD) to investigate the early-stage deformation (i.e., incipient micro-plasticity) of a small CuZr MG (5000 atoms) to understand how the temperature effect on elastic limit depends on individual, isolated microscopic ST events. Surprisingly, we observe an anomalous temperature effect in certain temperature ranges, where the elastic limit increases with increasing temperature. To overcome the limitations of the 1D PEL picture and quantitatively describe the ST events, we construct the energy-strain landscape (ESL) based on high-throughput strain-dependent minimum-energy path (MEP) calculations for the ST events sampled by the MD simulations. With the applied strain as a separate dimension from the reaction coordinate in the configuration space, the ESL clearly shows how these ST events compete with each other at different temperatures and external loadings. Through the ESL analysis, we show that the elastic limit anomaly is due to the irreversible-to-reversible transition of ST events with increasing temperature in the relevant time scales, controlled by a strain-independent property for each ST event called the ‘eigen barrier’. We further develop an analytic model predicting the elastic limit as a function of temperature under different strain rates. Additional MD simulations are performed to demonstrate that the predicted behavior is general by showing it occurs at larger sample sizes (20000 atoms) and different chemical species (NiNb).

## Results

### Stress–strain responses of Cu_64.5_Zr_35.5_ metallic glasses

**Fig. 2:**
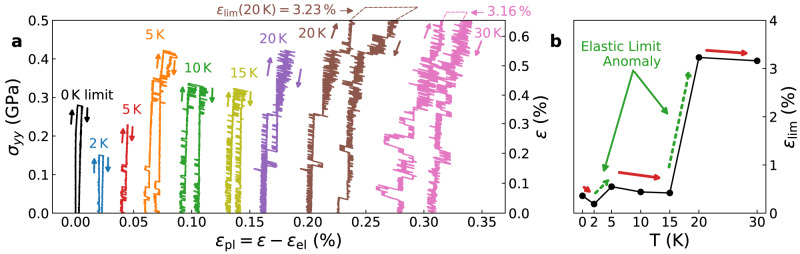
Anomalous temperature dependence of the elastic limit. **a** Stress–strain curves of the tensile loading tests at different temperatures in the *y*-direction up to the elastic limit (onset of inelasticity), then unloading back to zero stress. The elastic strain is removed from the total strain to reveal the ST events. Curves at non-zero temperatures are shifted to help with visualization. Two additional reversible cyclic loading tests are performed below the elastic limit at 5 K (the third curve, up to 0.3% strain) and 20 K (the seventh curve, up to 0.58% strain). **b** The simulated elastic limit ${\varepsilon }_{\lim }$ as a function of temperature. The solid (red) and dashed (green) arrows indicate the normal and anomalous temperature effects on ${\varepsilon }_{\lim }$.

We perform MD simulations of cyclic tensile loading-unloading tests (engineering strain rate $\dot{\varepsilon }=1{0}^{7}\,{{{{{{{{\rm{s}}}}}}}}}^{-1}$) on a well-annealed Cu_64.5_Zr_35.5_ MG sample (effective cooling rate 1.4 × 10^8^ K/s, see Methods) using LAMMPS^24^. Figure 2a shows the simulated stress-strain curves under the zero-temperature (i.e., athermal quasi-static) condition and a series of temperatures up to 30 K. All the simulations start from the same glassy state. The jumps on the loading curves indicate ST events – local atomic rearrangements that release strain energy. To reveal these jumps, we use the plastic strain *ε*_pl_ = *ε* − *ε*_el_ as the horizontal axis, where *ε* is the total strain, and *ε*_el_ = *σ*/*E* is the elastic strain with a modulus *E* = 77.5 GPa. The sample is unloaded back to zero stress right after each jump. The first jump resulting in a non-recoverable strain defines the elastic limit ${\varepsilon }_{\lim }$. ${\varepsilon }_{\lim }$ is generally expected to decrease with increasing temperature because the ST events are stress-driven thermally activated processes^25–27^. At a higher temperature, thermal activation can help overcome the higher energy barriers at a lower strain. However, as shown in Fig. 2b, while ${\varepsilon }_{\lim }$ decreases with increasing temperature in the ranges of 0 K–2 K, 5 K–15 K, and 20 K–30 K, it intermittently increases in the ranges of 2 K–5 K and 15 K–20 K. Similar anomalous (non-monotonic) temperature effects are observed in two independently prepared well-annealed Cu_64.5_Zr_35.5_ samples (Supplementary Fig. 3). However, the exact values of ${\varepsilon }_{\lim }(T)$ vary for different samples because the simulation cell is relatively small, and only a few ST events can be sampled. The following analysis is only performed on one of the three configurations (CuZr-3, See Supplementary Note 1).

### Strain dependence on the energy landscape

**Fig. 3:**
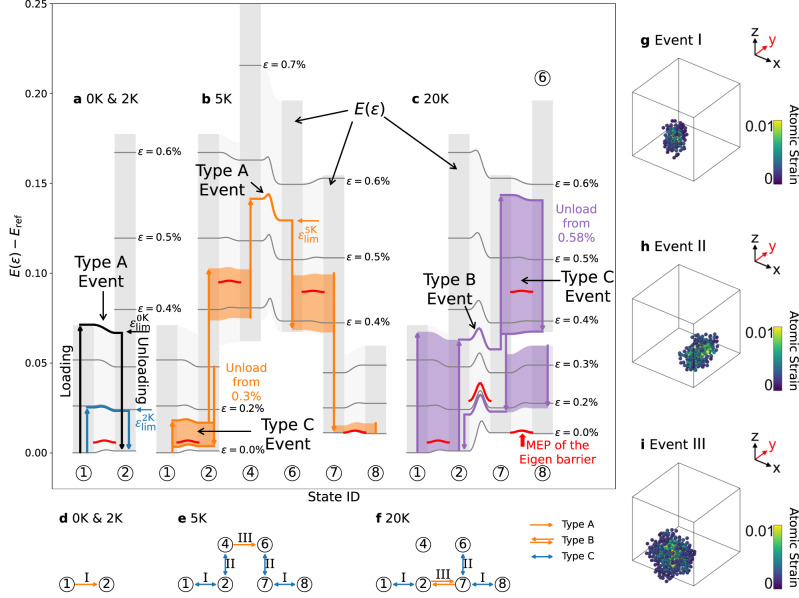
The energy-strain landscape (ESL) of the metastable state IDs (circled numbers) visited during loading-unloading cycles at different temperatures. **a** Quasi-static 0 K limit (loaded up to ${\varepsilon }_{\lim }=0.36\,\%$) and 2 K (loaded up to ${\varepsilon }_{\lim }=0.19\,\%$); **b** 5 K (loaded up to ${\varepsilon }_{\lim }=0.56\,\%$); **c** 20 K (loaded up to *ε* = 0.58%). Vertical gray bars represent the strain energy *E*(*ε*) of states as functions of strain *ε*, and the dark gray horizontal lines show the minimum-energy paths (MEPs) between neighboring states, illustrating the 1D PEL picture at applied strain *ε*. A reference elastic energy *E*_ref_(*ε*) = 1.5*ε*^2^ + *E*_0_ is subtracted from the total energy to magnify the energy differences, and *E*_0_ is the energy of the system at zero strain. Thick arrows indicate the energy change of the same state with strain during loading/unloading. Thick curves connecting the vertical arrows indicate the one-time transition between neighboring states, while shaded areas represent fully recoverable transitions. The red curves indicate the MEP of the eigen barrier for each shear transformation (ST) event. Three ST events (I, II, III) are involved in these MD simulations, and they experience three types of behavior: irreversible (Type A), hysteresis reversible (Type B), and fully reversible (Type C), marked out on the ESL. The connectivity between states and their types are illustrated at **d** 0 K limit and 2 K; **e** 5 K; **f** 20 K. The spatial extent of ST events I, II, and III are shown in **g**, **h**, and **i**, respectively.

To understand the origin of this anomalous increase of the elastic limit at 5 K and 20 K, we construct the energy-strain landscape (ESL) to shed light on the dynamics of the ST events during cyclic loading. Within the regime of early-stage deformation (*ϵ* < 0.6% strain) for this specific initial configuration, we find that three isolated ST events (Events I, II, and III) can completely describe the deformation behavior of the sample over the entire temperature range considered here. Our analysis starts with determining the inherent states (i.e., basins in PEL) by energy minimization from all the configurations periodically saved during the simulations (at every 0.0004% strain). We then use the maximum non-affine displacement matrix (MNADM)^28^ with the hierarchical clustering method^29^ to assign a state (i.e., basin) ID (circled numbers) to all the relaxed configurations (See Methods). An ST event is identified when two consecutive configurations are assigned different state IDs. Finally, we calculate the minimum-energy paths (MEP) of all the identified ST events as a function of strain by the nudged-elastic band (NEB) method (see Methods). Figure 3a-c show the ESL constructed along the state sequences identified in MD simulations at 0 K and 2 K, 5 K, and 20 K, respectively. The MEPs connecting neighboring states at different strains are represented as thin dark gray lines. Thick vertical arrows describe the energy change of the same state with strain during loading/unloading, and the thick lines connecting the arrows correspond to the transitions (i.e., ST events) between neighboring states.

### Temperature-dependent elastic limit and shear transformation (ST) events

At 0 K, the system stays in the initial State ① until 0.36% strain, where the energy barrier becomes zero for transition from ① to ②, as shown by the thick black line in Fig. 3a. The system stays in State ② during unloading without jumping back to ①. Hence, the elastic limit is ${\varepsilon }_{\lim }(0\,{{{{{{{\rm{K}}}}}}}})=0.36\%$. The transition from ① to ② is labeled as Event I, as shown in Fig. 3d, while the atoms involved in this ST event are shown in Fig. 3g. At 2 K, with the assistance of thermal fluctuation, Event I occurs earlier, at 0.19% strain, where the energy barrier remains non-zero, as shown by the blue curve in Fig. 3a. The system also stays in ② during unloading, and thus ${\varepsilon }_{\lim }(2\,{{{{{{{\rm{K}}}}}}}})=0.19\,\%$, lower than ${\varepsilon }_{\lim }(0\,{{{{{{{\rm{K}}}}}}}})$, as expected.

When the temperature increases further to 5 K, the transition from ① to ② starts to occur at even lower strains. However, due to the higher thermal fluctuation, the system now jumps back and forth between these two states over a range of strain, as indicated by a shaded orange region between ① and ② in Fig. 3b and a double arrow between the two states in Fig. 3e. The system then stays in State ② for a strain above 0.16%. If the sample is unloaded from a maximum strain of 0.30%, the system again jumps back and forth between States ② and ① once the strain drops below 0.16% and eventually returns to State ①. Because no residual strain is produced when the sample is fully unloaded, the elastic limit at 5 K must be higher than 0.30% strain. The actual ${\varepsilon }_{\lim }(5\,{{{{{{{\rm{K}}}}}}}})$ is 0.56%, which is higher than both ${\varepsilon }_{\lim }(0\,{{{{{{{\rm{K}}}}}}}})$ and ${\varepsilon }_{\lim }(2\,{{{{{{{\rm{K}}}}}}}})$, contrary to the common expectation. Therefore, the temperature dependence of the elastic limit depends not only on the strain at which an ST event is activated but also on whether that event is reversible at given temperatures.

Figure 3b shows how the elastic limit is reached at 5 K. When the loading strain exceeds 0.38%, both ST Event II and its reverse are activated, causing back-and-forth transitions between States ② and ④. Above strain 0.46%, the system stays in State ④ until ST Event III is activated at strain 0.56%, which brings the system to State ⑥. During unloading, Event II and its reverse become activated again over a strain range, followed by the activation of Event I and its reverse at lower strains. When the sample is fully unloaded, the effects of Events I and II are fully reversed, but the effect of Event III remains, resulting in a residual strain at 5 K. Hence, ${\varepsilon }_{\lim }(5\,{{{{{{{\rm{K}}}}}}}})=0.56\,\%$. Figure 3c shows that at 20 K, even Event III (which now occurs before Event II) can be reversed during unloading. As a result, the elastic limit at 20 K is no longer controlled by Event III, but by another ST event that gets activated at an even higher strain (3.23%).

From Fig. 3a–c, we can distinguish three types of reversibility behaviors for each ST event. A Type-A event is irreversible during the entire loading-unloading strain cycle (such as Event I at 0 K and Event III at 5 K). A Type-B event occurs during loading but reverts during unloading (such as Event III at 20 K), i.e., reversible with a hysteresis. A Type-C event and its reverse occur multiple times during loading and unloading (such as Events I and II at 5 K and 20 K), i.e., fully reversible. With increasing temperature, the same ST event can gradually change its type from A to B to C. Event I no longer dictates the elastic limit at 5 K because it has become fully reversible (Type-C). Similarly, Event III no longer dictates the elastic limit at 20 K because it has become reversible (Type-B). Therefore, the non-monotonic variation of ${\varepsilon }_{\lim }(T)$ in Fig. 2b can be well understood through the ESLs shown in Fig. 3a–c and the system’s trajectory in the state space shown in Fig. 3d–f. This analysis would not be possible using the one-dimensional PEL sketches such as Fig. 1b.

### Thermally activated irreversible-reversible transition of ST events

**Fig. 4:**
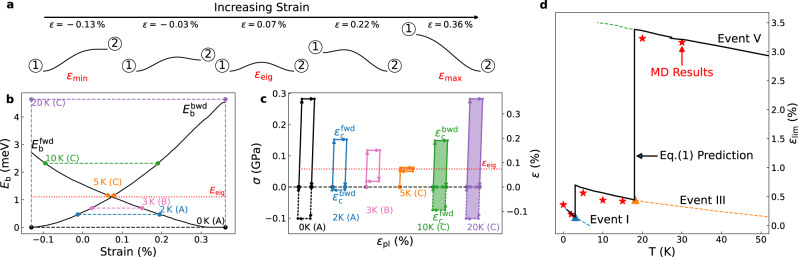
Predict elastic limit based on the reversibility of ST events. **a** Selected MEPs of the ST Event I at different applied strains within the range $({\varepsilon }_{\min },{\varepsilon }_{\max })$ where both States ① (initial state) and ② (final state) are metastable. **b** Energy barrier as functions of the applied strain *ε* for Event I in forward ${E}_{{{{{{{{\rm{b}}}}}}}}}^{{{{{{{{\rm{fwd}}}}}}}}}(\varepsilon )$ and backward ${E}_{{{{{{{{\rm{b}}}}}}}}}^{{{{{{{{\rm{bwd}}}}}}}}}(\varepsilon )$ directions. The thermal fluctuations at different temperatures are marked as horizontal dashed lines, and the eigen barrier *E*_eig_ is illustrated as the red dotted line. **c** Corresponding stress-strain hysteresis loops of Event I at different temperatures. *ε*_eig_ is the strain corresponding to the eigen barrier *E*_eig_. **d** Predicted elastic limit ${\varepsilon }_{\lim }(T)$ (solid black line) as a function of temperature and the simulated *ε*_l*im*_(*T*) from MD simulations (red stars).

We now examine what controls the critical temperature for an ST event to transition from irreversible (Type-A) to reversible (Type-B or C). Using Event I as an example, Fig. 4a shows its MEPs at different strains. Within the strain range $({\varepsilon }_{\min }=-\!0.13\%,{\varepsilon }_{\max }=0.36\%)$, where both States ① and ② are metastable, State ② (final state) is more stable at the higher strain while State ① (initial state) is more stable at the lower strain, consistent with previous simulation studies^25–27^. Correspondingly, the energy barrier in the forward direction ${E}_{{{{{{{{\rm{b}}}}}}}}}^{{{{{{{{\rm{fwd}}}}}}}}}$ decreases with increasing strain, while that in the backward direction ${E}_{{{{{{{{\rm{b}}}}}}}}}^{{{{{{{{\rm{bwd}}}}}}}}}$ increases, as shown in Fig. 4b. In the zero-temperature limit, Event I occurs only when ${E}_{{{{{{{{\rm{b}}}}}}}}}^{{{{{{{{\rm{fwd}}}}}}}}}$ drops to zero at the critical strain of ${\varepsilon }_{{{{{{{{\rm{c}}}}}}}}}^{{{{{{{{\rm{fwd}}}}}}}}}={\varepsilon }_{\max }$. Similarly, the Event would be reverted when ${E}_{{{{{{{{\rm{b}}}}}}}}}^{{{{{{{{\rm{bwd}}}}}}}}}$ drops to zero at the critical strain of ${\varepsilon }_{{{{{{{{\rm{c}}}}}}}}}^{{{{{{{{\rm{bwd}}}}}}}}}={\varepsilon }_{\min }$. Therefore, the ST event exhibits a hysteresis loop during the cyclic loading between the two critical strains $({\varepsilon }_{{{{{{{{\rm{c}}}}}}}}}^{{{{{{{{\rm{bwd}}}}}}}}},{\varepsilon }_{{{{{{{{\rm{c}}}}}}}}}^{{{{{{{{\rm{fwd}}}}}}}}})$, as shown in Fig. 4c. Since at 0 K, ${\varepsilon }_{{{{{{{{\rm{c}}}}}}}}}^{{{{{{{{\rm{bwd}}}}}}}}} \, < \, 0$, Event I does not revert after being unloaded to zero strain, i.e., it is an irreversible (Type-A) event.

The intersection of the ${E}_{{{{{{{{\rm{b}}}}}}}}}^{{{{{{{{\rm{fwd}}}}}}}}}$ and ${E}_{{{{{{{{\rm{b}}}}}}}}}^{{{{{{{{\rm{bwd}}}}}}}}}$ curves in Fig. 4a defines a particular strain where States ① and ② have the same energy, and the forward and backward barriers are equal, i.e., ${E}_{{{{{{{{\rm{b}}}}}}}}}^{{{{{{{{\rm{fwd}}}}}}}}}={E}_{{{{{{{{\rm{b}}}}}}}}}^{{{{{{{{\rm{bwd}}}}}}}}}$. We define the energy barrier at this specific strain as the eigen barrier ${E}_{{{{{{{{\rm{eig}}}}}}}}}\equiv {E}_{{{{{{{{\rm{b}}}}}}}}}^{{{{{{{{\rm{fwd}}}}}}}}}={E}_{{{{{{{{\rm{b}}}}}}}}}^{{{{{{{{\rm{bwd}}}}}}}}}$ and denote the corresponding strain as *ε*_eig_. When the thermal energy equals *E*_eig_, both forward and backward transitions would be activated at the same strain (*ε*_eig_) so that the size of the hysteresis loop shrinks to zero. It turns out that the thermal energy at 5 K is slightly above the eigen barrier *E*_eig_ for Event I. In Fig. 4b, this means that ${\varepsilon }_{{{{{{{{\rm{c}}}}}}}}}^{{{{{{{{\rm{fwd}}}}}}}}}$ is now lower than ${\varepsilon }_{{{{{{{{\rm{c}}}}}}}}}^{{{{{{{{\rm{bwd}}}}}}}}}$. Consequently, for strain in the range $({\varepsilon }_{{{{{{{{\rm{c}}}}}}}}}^{{{{{{{{\rm{fwd}}}}}}}}},{\varepsilon }_{{{{{{{{\rm{c}}}}}}}}}^{{{{{{{{\rm{bwd}}}}}}}}})$, the thermal energy is sufficient to overcome both the forward and backward transitions. As observed in the MD simulation, Event I and its reverse occur back-and-forth multiple times in this strain range, i.e., Event I has become fully reversible (Type-C).

### Prediction of reversibility and elastic limit based on rate theory

In summary, an ST event becomes Type-B if the thermal energy is above the backward energy barrier at zero strain, ${E}_{{{{{{{{\rm{b}}}}}}}}}^{{{{{{{{\rm{bwd}}}}}}}}}(\varepsilon=0)$, and becomes Type-C if the thermal energy is above the eigen barrier *E*_eig_; otherwise, the event is of Type-A. Based on the above understanding, we can now construct a theory to predict the temperature dependence of the elastic limit at any strain rate $\dot{\varepsilon }$ using the ST events’ energy barrier curves. At a given temperature *T*, recall that the elastic limit ${\varepsilon }_{\lim }(T)$ is the critical strain of the forward jump with the lowest ${\varepsilon }_{{{{{{{{\rm{c}}}}}}}}}^{{{{{{{{\rm{fwd}}}}}}}}}$ among all the accessible Type-A ST events. The critical strain ${\varepsilon }_{{{{{{{{\rm{c}}}}}}}}}^{{{{{{{{\rm{fwd}}}}}}}}}$ can be obtained from the energy barrier curve ${E}_{{{{{{{{\rm{b}}}}}}}}}^{{{{{{{{\rm{fwd}}}}}}}}}(\varepsilon )$ by solving the following implicit equation^30^ (see Supplementary Note 4).

1 $$\frac{{E}_{{{{{{{{\rm{b}}}}}}}}}^{{{{{{{{\rm{fwd}}}}}}}}}(\varepsilon )}{{k}_{{{{{{{{\rm{B}}}}}}}}}T}=\ln \frac{{k}_{{{{{{{{\rm{B}}}}}}}}}T\,{\nu }_{0}}{\dot{\varepsilon }\,\left|d{E}_{{{{{{{{\rm{b}}}}}}}}}^{{{{{{{{\rm{fwd}}}}}}}}}/d\varepsilon \right|}$$

where *k*_B_ is the Boltzmann constant, and *ν*_0_ = 3 × 10^11^ s^−1^ is the average vibrational frequency of the normal mode along the MEP direction in the initial state^30^. Similarly, the critical strain ${\varepsilon }_{{{{{{{{\rm{c}}}}}}}}}^{{{{{{{{\rm{bwd}}}}}}}}}$ for the reverse transformation can be obtained from the energy barrier curve ${E}_{{{{{{{{\rm{b}}}}}}}}}^{{{{{{{{\rm{bwd}}}}}}}}}(\varepsilon )$. Based on these solutions, the ST event becomes Type-B if ${\varepsilon }_{{{{{{{{\rm{c}}}}}}}}}^{{{{{{{{\rm{bwd}}}}}}}}} \, > \, 0$ and becomes Type-C if ${\varepsilon }_{{{{{{{{\rm{c}}}}}}}}}^{{{{{{{{\rm{bwd}}}}}}}}} \, > \, {\varepsilon }_{{{{{{{{\rm{c}}}}}}}}}^{{{{{{{{\rm{fwd}}}}}}}}}$.

The dashed lines in Fig. 4d show the prediction of ${\varepsilon }_{{{{{{{{\rm{c}}}}}}}}}^{{{{{{{{\rm{fwd}}}}}}}}}(T)$ for Events I, III, and V, all the Type-A events sampled during MD simulations at sufficiently low temperatures. The vertical lines in Fig. 4d indicate the temperature above which Event I or III are no longer a Type-A event, i.e., they no longer control the elastic limit. The black curve that connects the sections of the ${\varepsilon }_{{{{{{{{\rm{c}}}}}}}}}^{{{{{{{{\rm{fwd}}}}}}}}}(T)$ curves where the events remain Type-A predicts the elastic limit as a function of temperature. The theoretical prediction of ${\varepsilon }_{\lim }(T)$ is in excellent agreement with the observations from MD simulations (red stars). It is worth noting that the reversibility of an event also depends on the time scale of observation, and hence the strain rate. When the cyclic loading is performed at a substantially lower strain rate, an irreversible (Type-A) ST event has more waiting time to attempt the reverse jump so that it can become a reversible event at a lower critical temperature than at a higher strain rate. With a decreasing strain rate, the predicted transition temperature (where the elastic limit jumps) shifts towards lower temperatures, as shown in Supplementary Fig. 8.

## Discussion

Here we have shown that the ST events can be identified from the energy landscape analysis of MD trajectories. There is also significant interest in finding good spatial (geometrical) indicators that can be used to identify potential sites for ST events, such as free volume^31^, short-range order^32^, and radial distribution function^33^. Thus, we calculate the shear-transformation zone (STZ) size and free volume (FV) changes for each ST event (I, II, and III) sampled in the MD simulations as a function of strain (Supplementary Fig. 9). We find that the STZ sizes of different ST events, in general, are proportional to the eigen barrier (Supplementary Fig. 10), but these geometric indicators are not sensitive to the applied strain for a given event. At the same time, the energy barrier of an ST event has a strong dependence on the applied strain, which (as we have shown) has significant consequences on the inelastic behavior of MGs. Therefore, we do not expect the geometric features of STZ to correlate well with the energy barrier at arbitrary strain. These findings further illustrate the significance of the eigen barrier as an intrinsic strain-independent property of an ST event.

Because the time scale of MD simulations is much shorter than the experimental time scale, the simulated cooling rate for the sample preparation is limited to several orders of magnitude higher than experimental conditions. To mitigate this time-scale problem, we use the annealing method proposed by Zhang et al. (2015)^34^, and reach an effective cooling rate of 1.4 × 10^8^K/s. The diffusion coefficient of our annealed configuration (CuZr-3) is *D* ~ 10^−13^ m^2^/s at 700 K, consistent with previous reports^35,36^. Admittedly, this value is still much higher than the experimental range^37^ of 10^−22^ m^2^/s-10^−17^ m^2^/s, a consequence of the high cooling rate. However, compared with existing atomistic simulations, our cooling rate and diffusivity are still state-of-the-art. To demonstrate that our prediction of the anomalous temperature dependence of the elastic limit is insensitive to the diffusion coefficient, we perform another set of cyclic loading simulations on the same configuration subjected to a confining pressure of ~ 10 GPa (CuZr-compressed). This confining pressure induces a  4% compressive strain, leading to a reduction of free volume and about an order- of-magnitude reduction of the diffusion coefficient *D*, as shown in Supplementary Fig. 4a. In addition, a recent study^38^ applied a mixed Molecular Dynamics - Monte Carlo algorithm to generate a well-relaxed Cu_50_Zr_50_ metallic glass configuration with an effective cooling rate of 500 K/s. MD simulations of this slow-cooling configuration (CuZr-R) show a diffusivity of ~ 1 × 10^−15^ m^2^/s, two orders of magnitude lower than our annealed configuration (Supplementary Fig. 4a). For all three configurations, although with different diffusivity, the predicted non-monotonic temperature dependence of the elastic limit is clearly visible at these low-diffusivity conditions (CuZr-compressed and CuZr-R), as shown in Supplementary Fig. 4b, c. This result confirms that the predicted behavior is general and is likely to occur for configurations with even lower diffusivity.

To assess the generality of the predicted anomalous temperature effects on the elastic limit, we repeat the cyclic loading on two independently prepared CuZr configurations with 5000 atoms, a larger CuZr sample with 20000 atoms (Supplementary Fig. 4d), and two Ni_60_Nb_40_ samples with 5000 atoms (Supplementary Text II). The resulting ${\varepsilon }_{\lim }(T)$ reproduces a similar non-monotonic trend as Fig. 2b for all these samples, i.e., intermittent increases of ${\varepsilon }_{\lim }$ with increasing temperature (see Supplementary Fig. 3). It is worth further studies using larger-scale MD simulations to obtain the collective behavior of more ST events in response to the applied loading. Due to the difficulty of low-temperature mechanical tests, the elastic limit measurements were rarely reported for MGs below 20 K. Interestingly, existing failure stress measurements of Fe_40_Ni_40_P_14_B_6_ and Pd_84.5_Si_15.5_ MGs^39^ indeed show a non-monotonic temperature dependence in the temperature range of 0.5 K–4.2 K. Although some authors attributed this anomalous behavior to quantum effects^2^, we suggest that this non-monotonic temperature dependence could be the result of the transition of ST events from irreversible (Type-A) to reversible (Type-B or C) with increasing temperature. We have demonstrated that the energy-strain landscape (ESL) can be used to clarify the atomistic mechanisms of incipient plastic deformation in MGs. We expect that the ESL framework can be combined with other advanced sampling methods^13,40,41^ to elucidate the mechanisms of interaction and coalescence of ST events that lead to shear band formation and failure of MGs.

## Methods

### Molecular dynamics simulations

We prepare a Cu_64.5_Zr_35.5_ sample of 5000 atoms with a cubic simulation box of size 43 × 43 × 43 Å. Periodic boundary conditions are imposed in all directions to simulate the bulk system. We use the LAMMPS package^24^ to perform MD simulations with the embedded-atom model (EAM) developed by Mendelev et al.^42^ to study mechanical properties of CuZr metallic glasses. The initial glass configuration is obtained by the following cooling processes with NPT ensemble at zero pressure: A 2000 K Cu_64.5_Zr_35.5_ liquid is directly cooled down to 700 K at a constant cooling rate of 1 × 10^10^ K s^−1^, and the system is then annealed at 700 K for 2 μs. The annealed configuration is further quenched to 300 K at a constant cooling rate of 1 × 10^10^ K ⋅ s^−1^, followed by 1 ns of annealing at 300 K to obtain the initial configuration for loading tests. The effective cooling rate can be determined as 1.4 × 10^8^ K s^−1^ by extrapolating the system energies (at 300 K) of direct MD simulations at higher cooling rates (10^10^–10^14^ K s^−1^)^34^ (Supplementary Note 1).

The annealed initial configuration is deformed under uniaxial tensile loading up to the engineering strain of 8% in the *y*-direction at different temperatures using NVT ensemble. The tensile strain is applied by extending the simulation box at a constant strain rate of 10^7^*s*^−1^ with a constant Poisson’s ratio of 0.4 and remapping the atoms’ positions to the simulation box at every timestep. The 0 K simulation is performed by athermal quasi-static simulation, where the configuration is deformed by 0.0004% strain increments followed by energy minimization. The loaded configuration after each jump on the stress-strain curves is unloaded back to zero stress with the same strain rate 10^7^ s^−1^. The residual strain after unloading is used to determine whether the jump is reversible and whether the elastic limit is reached.

Three distinct Cu_64.5_Zr_35.5_ configurations are prepared and deformed independently using the same MD settings as described above. One of the three configurations (CuZr-3, Supplementary Note 2) is analyzed with the following methods, leading to the conclusion of this study.

### Determining the metastable states

To determine the metastable state visited during MD simulations and assign state IDs to the saved configurations, we calculate the maximum non-affine distance matrix (MNADM), presenting the maximum atom displacements between every pair of energy-minimized configurations at different strains:

2 $${\Delta }^{\max }({\varepsilon }_{1},{\varepsilon }_{2})=\mathop{\max }\limits_{i}{\left|{{{{{{{{\bf{r}}}}}}}}}_{i}({\varepsilon }_{1})-{{{{{{{\bf{H}}}}}}}}({\varepsilon }_{1})\cdot {{{{{{{{\bf{H}}}}}}}}}^{-1}({\varepsilon }_{2})\cdot {{{{{{{{\bf{r}}}}}}}}}_{i}({\varepsilon }_{2})\right|}_{2}$$

where **r**_*i*_ is the atom position of the atom *i*. ${{{{{{{\bf{H}}}}}}}}(\varepsilon )=\left[{{{{{{{{\bf{c}}}}}}}}}_{1},{{{{{{{{\bf{c}}}}}}}}}_{2},{{{{{{{{\bf{c}}}}}}}}}_{3}\right]$ is the simulation cell matrix at strain *ε*, and {**c**_1_, **c**_2_, **c**_3_} are the edge vectors of the simulation cell. **H**(*ε*_1_) ⋅ **H**^−1^(*ε*_2_) represents the affine-transformation tensor that removes the homogeneous cell deformation between the strains *ε*_1_ and *ε*_2_. The maximum atom displacement is used instead of the average displacement because the maximum is more sensitive to isolated ST events containing only a few atoms.

We treat each energy-minimized configuration as a node in a graph, and an edge between two nodes is the similarity between the two configurations defined by the MNADM. The hierarchical clustering method is applied to cluster the configurations into groups. Each group corresponds to a metastable state on the PEL (Supplementary Note 3, Supplementary Fig. 5). A minimum distance of 0.1 Å is selected as the input parameter to the clustering algorithm, meaning that any node within a cluster has at least one other node in the same cluster with a distance below 0.1 Å. The MNADM is also used to confirm the elastic limit determined from the residual strain in the previous section (Supplementary Note 3, Supplementary Fig. 6).

### Construct the energy-strain landscape (ESL)

After the inherent states are determined, we use the nudge-elastic band (NEB) method to calculate the minimum-energy paths (MEP) and their strain dependence. For an ST event occurring at strain *ε*_ST_ (Supplementary Fig. 7), the configuration before the event is State ①, and the configuration after the event is State ②. Athermal quasi-static (0 K) loading and unloading are applied to obtain State ① and ②’s configurations at different strains, within the strain range of $({\varepsilon }_{\min },{\varepsilon }_{\max })$ where both states are stable. Finally, we apply a series of NEB calculations between configurations of States ① and ② at different strains to obtain the strain-dependent MEP of the ST event. The MEPs of the same ST event are stacked into an additional dimension of strain to construct the energy-strain landscape shown in Fig. 3a–c. A reference elastic energy *E*_ref_(*ε*) = 1.5*ε*^2^ + *E*(*ε* = 0) is subtracted from the total energy to reveal the energy drops induced by ST events.

### Supplementary information

Supplementary Information

### Source data

Source Data Files

## Data Availability

The simulation data in this study are available on GitLab, MG_ElastLim repository^43^ (10.5281/zenodo.10184745). The raw data for making the plots in the manuscript is included in the Supplementary Data. Source data are provided in this paper.

## Code availability

The simulation and analysis scripts in this study are available on GitLab, MG_ElastLim repository^43^ (10.5281/zenodo.10184745).
