# Supplementary material for: Anomalous temperature dependence of elastic limit in metallic glasses
Source: Nat Commun. 2024 Jan 2;15:171. doi: 10.1038/s41467-023-44048-7 (PMC10761975; doi:10.1038/s41467-023-44048-7)
Supplement: Supplementary file 1 — Supplementary Information [file 41467_2023_44048_MOESM1_ESM.pdf]

Supplementary Information for Anomalous Temperature Dependence of Elastic Limit in Metallic Glasses

Yifan Wang[1], Jing Liu[2], Jian-Zhong Jiang[2,3*], Wei Cai[1*]

[1*]Department of Mechanical Engineering, Stanford University, Stanford, 94305, CA, USA.

[2]International Center for New-Structured Materials (ICNSM), and School of Materials Science and Engineering, Zhejiang University, Hangzhou, 310027, Zhejiang, P.R. China.

[3]School of Materials Science and Engineering, Fuyao University of Science and Technology, Fuzhou, Fujian, P.R. China.

*Corresponding author(s). E-mail(s): jiangjz@zju.edu.cn; caiwei@stanford.edu;
Contributing authors: yfwang09@stanford.edu; liujinger@zju.edu.cn;

Supplementary Note 1. Preparation of well-annealed configurations. We prepare the $Cu_{64.5}Zr_{35.5}$ metallic glass (MG) configuration of 5000 atoms with a cubic simulation box of $43\,\text{Å} \times 43\,\text{Å} \times 43\,\text{Å}$ and periodic boundary conditions. Molecular dynamics (MD) simulations are performed using the LAMMPS package [1] and the embedded-atom model (EAM) developed by Mendelev et al. [2] as the interatomic potential. All the cooling simulations are performed under the NPT ensemble to ensure zero external stress, and the timestep is selected to be 2.5 fs. The well-annealed configuration is prepared with the following cooling-annealing schedule (Supplementary Fig. 1), with an effective cooling rate [3] of $1.4 \times 10^8\,\text{K} \cdot \text{s}^{-1}$.

The cooling schedule for the well-annealed configuration is shown as the blue line in Supplementary Fig. 1a. The temperature of the MG configuration is first raised to 2000 K to obtain a liquid configuration. We then directly quench the liquid configuration to 700 K with a cooling rate of $10^{10}\,\text{K} \cdot \text{s}^{-1}$, right below the glass transition temperature ($T_g \approx 750\,\text{K}$) for $Cu_{64.5}Zr_{35.5}$ MG [2]. The configuration is then annealed at 700 K for 2 μs to reduce the system's energy. We then cool down the annealed configuration to 300 K with the same cooling rate ($10^{10}\,\text{K} \cdot \text{s}^{-1}$), followed by 1 ns of annealing at 300 K. The system's energy (blue dots in Supplementary Fig. 1b) is determined by averaging the total energy over the 1 ns 300 K-annealing process. This energy is used to determine the effective cooling rate of the configuration. The 300 K-configuration is further cooled down to 2 K with the same cooling rate ($10^{10}\,\text{K} \cdot \text{s}^{-1}$) to obtain the final configuration (blue star in Supplementary Fig. 1a) for the tensile test at low temperatures.

To determine the effective cooling rate, we create reference configurations by uniformly cooling the liquid state from 2000 K to 300 K with five different cooling rates of $10^{14}, 10^{13}, 10^{12}, 10^{11}$, and $10^{10}\,\text{K} \cdot \text{s}^{-1}$, followed by 1 ns annealing at 300 K (black lines in Supplementary Fig. 1a). The reference system's energy is obtained from the averaged atomic energy during the 300 K-annealing process (black crosses in Supplementary Fig. 1b). Each cooling rate is repeated three times independently. The effective cooling rate (blue dots in Supplementary Fig. 1b) is determined as $1.4 \times 10^8\,\text{K} \cdot \text{s}^{-1}$ by extrapolating the linear fit (red dashed line) of the brute-force cooling simulations (black crosses).

To assess the generality of the conclusions, we also prepared $Ni_{60}Nb_{40}$ metallic glass configurations using the EAM potential developed by Zhang et al. [4]. The $Ni_{60}Nb_{40}$ configurations are produced with direct cooling of a liquid structure from 2000 K to 2 K at a constant cooling rate of $10^9\,\text{s}^{-1}$.

Supplementary Note 2. MD simulations of cyclic tensile loading tests for determining elastic limits. In order to obtain the initial configuration for the cyclic tensile loading test, the well-annealed configuration from the previous section is first equilibrated at the target temperature (2 K, 5 K, 10 K, 15 K, 20 K, and 30 K) for 1 ns using the NPT ensemble to ensure zero external stress. The configuration is further equilibrated for another 1 ns using the NVT ensemble with the average simulation cell during the NPT-equilibrium process. The equilibrated configuration is then ready for the tensile test.

The cyclic tensile test is performed under the NVT ensemble to avoid stress fluctuations. We apply uniaxial loading up to 8 % engineering strain in the y-direction with a constant strain rate of $10^7\,\mathrm{s}^{-1}$ by continuously changing the box size at every timestep, with a constant Poisson's ratio of 0.4. After each shear-transformation (ST) event (jumps on the stress-strain curve), the configuration is unloaded to zero stress with the same strain rate ($10^7\,\mathrm{s}^{-1}$). The first ST event with non-recoverable strain is defined as the elastic limit $\varepsilon_{\mathrm{lim}}$, schematically shown as curve (iv) in Supplementary Fig. 2. The configurations are saved at every 0.0004 % engineering strain, followed by energy minimization to obtain the corresponding metastable inherent state (IS). The cyclic tensile loading simulation at the 0 K limit is performed using molecular statics: the simulation box is uniaxially loaded by 0.0004 % strain increments in the y-direction (Poisson's ratio of 0.4), followed by energy minimization. The actual $\varepsilon_{\mathrm{lim}}$ is rounded-up to two significant digits considering the randomness of the thermally activated ST events.

We perform the cyclic tensile tests and determine $\varepsilon_{\mathrm{lim}}(T)$ of independently prepared MG configurations. Four $Cu_{64.5}Zr_{35.5}$ configurations (CuZr-1, CuZr-2, CuZr-3, and CuZr-20000) are prepared for examining the reproducibility of the conclusions. CuZr-1, CuZr-2, and CuZr-3 are the small configurations with 5000 atoms, and CuZr-20000 is the large configuration with 20000 atoms. CuZr-3 is the configuration examined in the main text. To test the generality of the conclusions, we also prepared two $Ni_{60}Nb_{40}$ configurations NiNb-1 and NiNb-2, with 5000 atoms. Supplementary Fig. 3 shows the stress-strain curves of CuZr-1, CuZr-2, NiNb-1, and NiNb-2. Supplementary Fig. 4d shows the stress-strain curves of CuZr-20000 configuration. The horizontal axis of the stress-strain curve uses the plastic strain $\varepsilon_{\mathrm{pl}} = \varepsilon - \varepsilon_{\mathrm{el}}$ to better reveal the jumps. The elastic strain $\varepsilon_{\mathrm{el}} = \sigma/E$ is removed from the total strain with Young's modulus $E = 77.5\,\mathrm{GPa}$ for $Cu_{64.5}Zr_{35.5}$ ($E = 83.5\,\mathrm{GPa}$ for $Ni_{60}Nb_{40}$). The anomalous non-monotonic temperature dependence of the elastic limit is readily seen for all the independently prepared samples, indicating the generality of our conclusions in the main text for glassy materials.

Metallic glasses are materials at metastable states. The internal local structures of metallic glasses are closely related to the energy and diffusivity [5], where the sample with slower cooling rate (longer annealing time) tends to move to metastable states with lower energy and lower diffusivity. By showing that our conclusion of the non-monotonic trend is decoupled from diffusion coefficient D, we demonstrate that our predictions of the anomalous temperature dependence of the elastic limit do not depend on specific internal structures. We perform cyclic loading simulations on the same CuZr-3 configuration subjected to a confining pressure of $\sim 10\,\mathrm{GPa}$, enforcing

an order of magnitude reduction of the diffusion coefficient D (See Supplementary Fig. 4a). Supplementary Fig. 4b shows the MD results of the temperature dependence of the elastic limit, confirming that the predicted anomalous temperature dependence is a general behavior likely to occur for samples with lower diffusivity, such as the ones prepared by lower cooling rates. In addition, we use the slow-cooling configuration CuZr-R obtained by mixed Molecular Dynamics and Monte Carlo methods with an effective cooling rate of $500\,\mathrm{K}\cdot\mathrm{s}^{-1}$ [6]. The corresponding diffusivity D is two orders of magnitude lower than compressed CuZr-3, showing the configuration is well relaxed, shown in Supplementary Fig. 4a as the green curve. Supplementary Fig. 4c shows the MD results of the temperature dependence of the elastic limit for the slow-cooling CuZr-R, further confirming that the non-monotonic temperature dependence of the elastic limit is a general behavior.

Supplementary Note 3. Determine the metastable inherent states (ISs) and shear-transformation (ST) events. Supplementary Fig. 5a illustrates the stress-strain curve of the cyclic loading simulation up to 0.4 % strain at 2 K. One ST event is observed during loading. After the ST event has occurred, the event is not reverted after unloading. There are 2000 relaxed configurations (1000 for loading and 1000 for unloading) saved during the simulation, and the corresponding MNADM includes 2000×2000 entries, as shown in Supplementary Fig. 5b. The color in the MNADM indicates the distance between configurations at different strains ε_1 and ε_2. It is readily seen that configurations of the same state have small distances, while the distances between different states are significant. It is found that the system undergoes States ① and ② during the cyclic loading. An unrecoverable ST event occurs at the strain of 0.19 %, which cannot be reverted after unloading, defined as the elastic limit $\varepsilon_{\mathrm{lim}}$.

We calculate the MNADM and perform clustering analysis on the cyclic loading test at 5 K, as illustrated in Supplementary Fig. 6a. For the cyclic loading up to 0.3 % strain, it is readily seen that the distance between the configurations before loading and after unloading (upper left or lower right corner) is small, as shown in Supplementary Fig. 6b, which means that these two configurations belong to the same state, and all the ST events have recovered after unloading. It is readily seen that the system jumps back and forth between States ① and ②. The ST event between States ① and ② no longer defines the elastic limit. For the cyclic loading test up to 0.6 % strain, the ST event corresponding to the plastic deformation is not recovered after unloading, indicating that the elastic limit $\varepsilon_{\mathrm{lim}}$ is at strain 0.56 %, as shown in Supplementary Fig. 6c. Similarly, at 20 K, the cyclic loading up to 3.0 % strain is reversible, while loading up to 3.5 % produces unrecoverable plastic deformation, indicating an elastic limit of 3.23 %, as shown in Supplementary Fig. 6d-f.

Supplementary Note 4. Implicit expression for predicting ST events at finite temperatures. Zhu et al. [7] derived the implicit expression for the stress to activate a stress-driven thermally activated process under constant temperature T and strain rate $\dot{\varepsilon}$,

$$\frac{G_{\mathrm{c}}(\sigma,T)}{k_{\mathrm{B}}T} = \ln\left[\frac{k_{\mathrm{B}}T\,N\nu_0}{E\dot{\varepsilon}\Omega_{\mathrm{c}}(\sigma,T)}\right] \tag{1}$$

where k_{B} is the Boltzmann constant, and E is the Young's modulus. The number of nucleation sites in our case $N = 1$. $G_{\mathrm{c}}(\sigma,T)$ is the activation free energy at constant stress σ and temperature T, and $\Omega_{\mathrm{c}}(\sigma,T) = -(\partial G_{\mathrm{c}}/\partial\sigma)_T$ is the activation volume. The activation Gibbs free energy $G_{\mathrm{c}}(\sigma,T)$ at a given stress σ is equivalent to the Helmholtz free energy $F_{\mathrm{c}}(\varepsilon,T)$ at the corresponding strain $\varepsilon(\sigma,T)$, i.e., $G_{\mathrm{c}}(\sigma,T) = F_{\mathrm{c}}(\varepsilon,T)$ [8]. In this work, under sufficiently low-temperature conditions, the entropic effects [9] can be neglected, and the free energy is reduced to the zero-temperature energy barrier $E_{\mathrm{b}}(\varepsilon)$ defined by the MEP, i.e., $G_{\mathrm{c}}(\sigma,T\approx 0) = F_{\mathrm{c}}(\varepsilon,T\approx 0) \approx E_{\mathrm{b}}(\varepsilon)$.

During the early-deformation stage, the stress-strain relation is very close to the linear response, i.e., $\sigma = E\varepsilon$. Therefore, the activation volume can also be estimated from $E_{\mathrm{b}}(\varepsilon)$:

$$E\Omega_{\mathrm{c}}(\sigma,T\to 0) = -\left[\frac{\partial G_{\mathrm{c}}}{\partial(\sigma/E)}\right]_{T\to 0} = -\left(\frac{\mathrm{d}E_{\mathrm{b}}}{\mathrm{d}\varepsilon}\right) \tag{2}$$

which leads to (equation (1) in the main text),

$$\frac{E_{\mathrm{b}}^{\mathrm{fwd}}(\varepsilon)}{k_{\mathrm{B}}T} = \ln\frac{k_{\mathrm{B}}T\,\nu_0}{\dot{\varepsilon}\,\left|dE_{\mathrm{b}}^{\mathrm{fwd}}/d\varepsilon\right|} \tag{3}$$

The vibrational frequency is determined as $\nu_0 = (2\pi)^{-1}\sqrt{Km^{-1}}$, where $m = (0.645m_{\mathrm{Cu}}^{-1} + 0.355m_{\mathrm{Zr}}^{-1})^{-1}$ is the average atomic mass, and K is the curvature of the initial basin along the MEP direction. We use a constant value $\nu_0 = 3\times 10^{11}\,\mathrm{s}^{-1}$, which is an average vibrational frequency obtained from the NEB calculations.

Supplementary equation (3) accounted for the reversibility of the ST events depending on the time scale (strain rate) of interest. An event that appears irreversible at a specific time scale may appear reversible at a much longer time scale, i.e., the reverse jump may occur if we wait for a very long time, or equivalently, when the loading/unloading strain rate is low. Supplementary Fig. 8 shows the predicted elastic limit at different loading strain rates. At a lower strain rate, an irreversible ST event would have more waiting time so that it can become reversible. Therefore, the non-monotonic anomalous temperature behavior would have a shorter temperature range compared to the high strain rate case. At a typical experimental strain rate of $10^{-2}\,\mathrm{s}^{-1}$, the anomalous temperature dependence of the elastic limit shrinks down to below 5 K. The experimental mechanical test is challenging at such low temperatures.

Supplementary Note 5. Spatial analysis: shear-transformation zone (STZ) and free volume (FV). Besides the energy barrier calculations, we further perform spatial analyses on the three ST events (I, II, III) during the early-stage deformation to examine whether there is any strain dependence on the structural indicators, including the shear-transformation zone (STZ) and free volume (FV). Supplementary Fig. 9a shows the forward and backward energy barrier curves for the three events (I, II, and III) discussed in the main text. The eigen barrier E_{eig} for Events I, II, and III marked by solid spheres in Supplementary Fig. 9a are estimated to be 1.102, 0.617, and 9.855 meV, respectively.

To determine the atoms involved in each ST event, we use the von Mises atomic shear strain η_{Mises} implemented in OVITO [10, 11], calculated based on the initial and final configurations in the MEPs obtained from the abovementioned NEB calculations. We analyzed the three ST events (I, II, III) discussed in the main text, and the STZ is defined as a cluster of atoms with $\eta_{\mathrm{Mises}} > 0.01$. About 70 atoms are involved as event III occurs, while less than 20 participate in the atomic rearrangements during events I and II, as shown in Supplementary Fig. 9b.

The free volume (FV) is calculated as the percolating volumes between atoms within a spherical region with radius $r_{\mathrm{c}} = 4.5\,\text{Å}$ centered at the STZ center-of-mass. Here we consider the Cu and Zr atoms as hard spheres with radii of 1.28 Å and 1.6 Å, respectively. The FV change as a function of strain for the three ST events is illustrated in Supplementary Fig. 9c. Similar trends were obtained while using other values $r_{\mathrm{c}} = 4$, 5, and 5.5 Å.

Supplementary Fig. 10 shows the STZ size over the eigen barrier of ST events (round dots) and the linear fitting (dashed line), indicating the higher the eigen barrier of a ST event, the larger the STZ size in MGs.

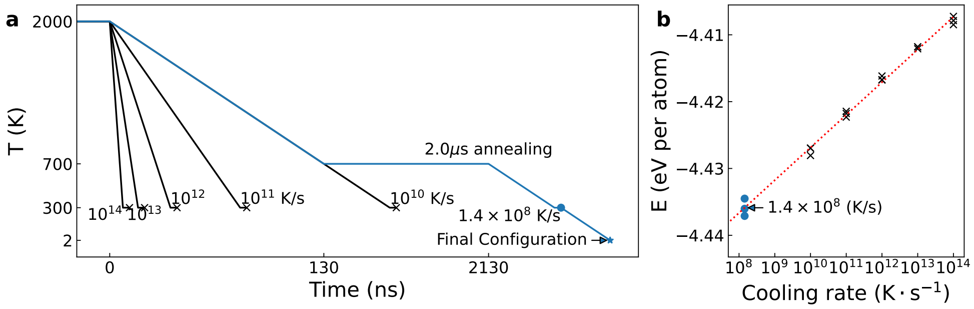

Supplementary Fig. 1 Preparing the slow-cooling configuration. **a** Cooling schedule for preparing the initial configuration at different cooling rates. **b** Extrapolation to determine the cooling rate.

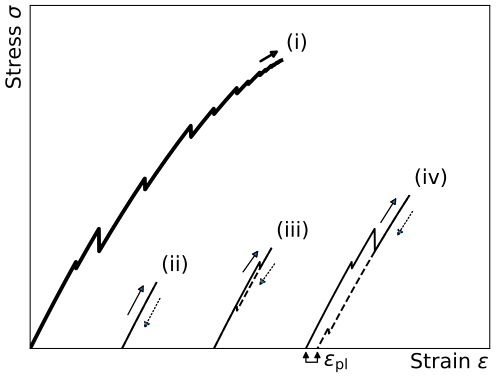

Supplementary Fig. 2 Schematic stress-strain curves of strain-control uniaxial loading tests. The curves (ii)(iii)(iv) are shifted to help with visualization. (i) uniaxial loading to the maximum strain, the jumps on the stress-strain curves represent shear transformation (ST) events. (ii) the uniaxial cyclic loading-unloading test within the elastic regime. (iii) the uniaxial cyclic loading test with a reversible ST event. (iv) indicates the elastic limit, the first irreversible ST event during the uniaxial cyclic test with non-recoverable strain after unloading.

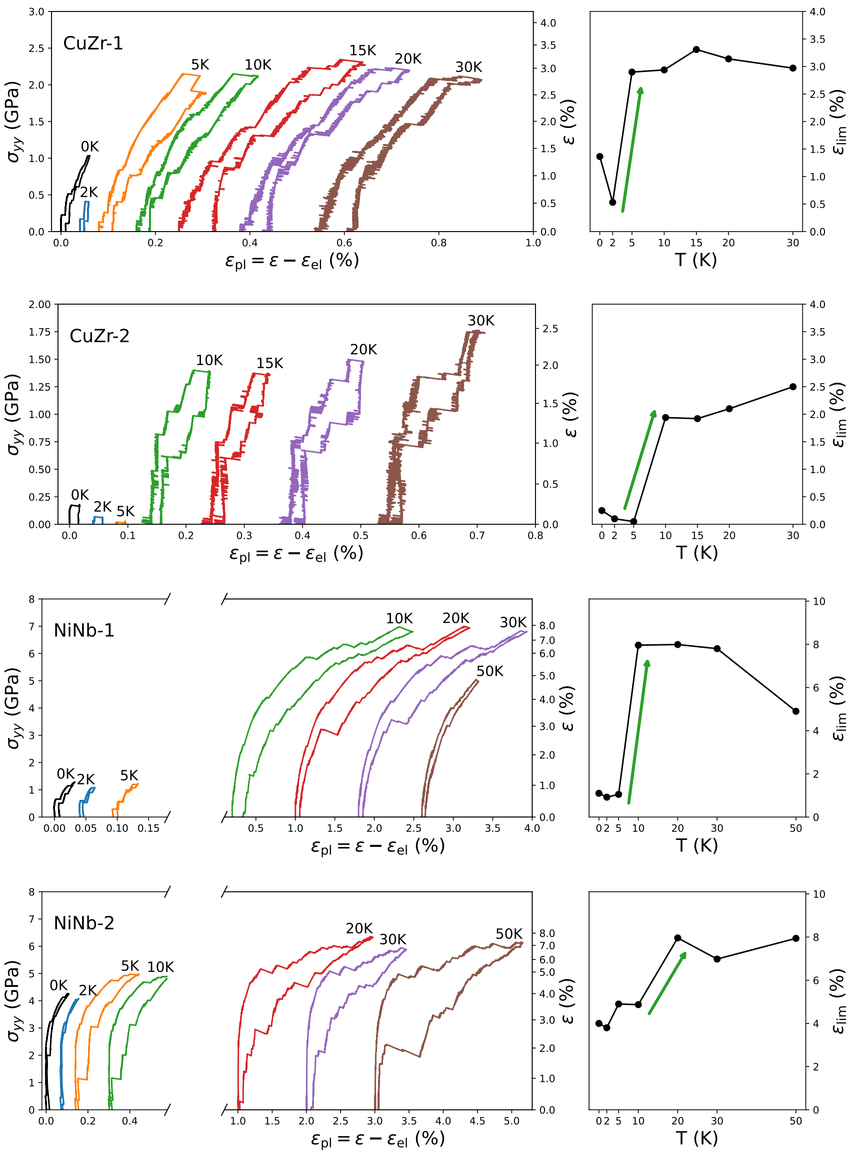

Supplementary Fig. 3 Onset inelasticity of four independently prepared metallic glass (MG) configurations. Stress-strain curves of cyclic tensile loading tests and the temperature dependence of elastic limit $\varepsilon_{\mathrm{lim}}(T)$ for the four independently prepared MG configurations (two $Cu_{64.5}Zr_{35.5}$ and two $Ni_{60}Nb_{40}$), named as CuZr-1, CuZr-2, NiNb-1, and NiNb-2, respectively. The curves are shifted rightwards on the horizontal axis for better visualization. The elastic limits for all samples show anomalous non-monotonic trends (green arrow) with increasing temperature.

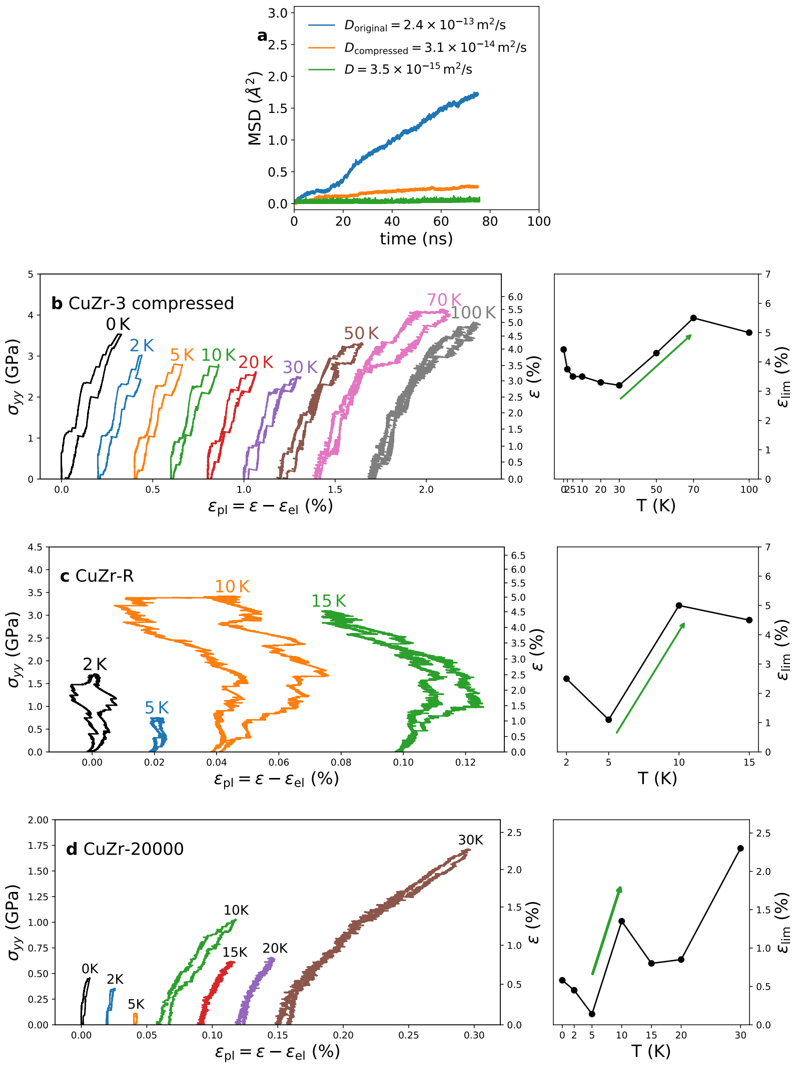

Supplementary Fig. 4 Onset inelasticity for the low-diffusivity configurations. **a** Mean-square displacement at 700 K and the fitted diffusion coefficient of the original (CuZr-3) configuration the compressed (with hydrostatic pressure of ~ 10 GPa) CuZr-3 configuration, and slow-cooling $Cu_{50}Zr_{50}$ configuration (CuZr-R) from Zhang et al. (2022) [6], with $500\,\mathrm{K \cdot s^{-1}}$ effective cooling rate. **b** Stress-strain curves of cyclic tensile loading tests and the temperature dependence of elastic limit $\varepsilon_{\rm lim}(T)$ for the compressed CuZr-3 configuration, **c** $Cu_{50}Zr_{50}$ configuration (CuZr-R) with 14400 atoms and **d** $Cu_{64.5}Zr_{35.5}$ configuration with 20000 atoms (CuZr-20000). The elastic limit shows a similar anomalous non-monotonic trend (green arrow) with increasing temperature.

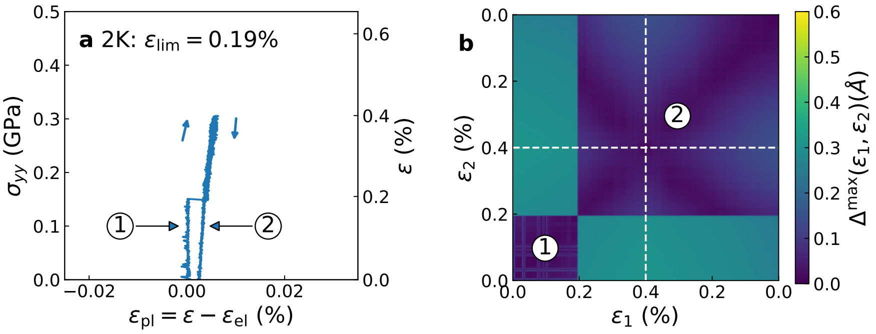

Supplementary Fig. 5 Determining metastable states using maximum non-affine distance matrix (MNADM). **a** Stress-strain curve of the 2 K cyclic loading simulation up to 0.4 % strain. The elastic limit is the first unrecoverable ST event from State ① to State ②. **b** Corresponding MNADM $\Delta_{\max}(\varepsilon_1, \varepsilon_2)$ (size 2000×2000). The white dashed line separates the loading and unloading stages of the cyclic loading. The dark blocks along the diagonal are labeled by their corresponding state numbers.

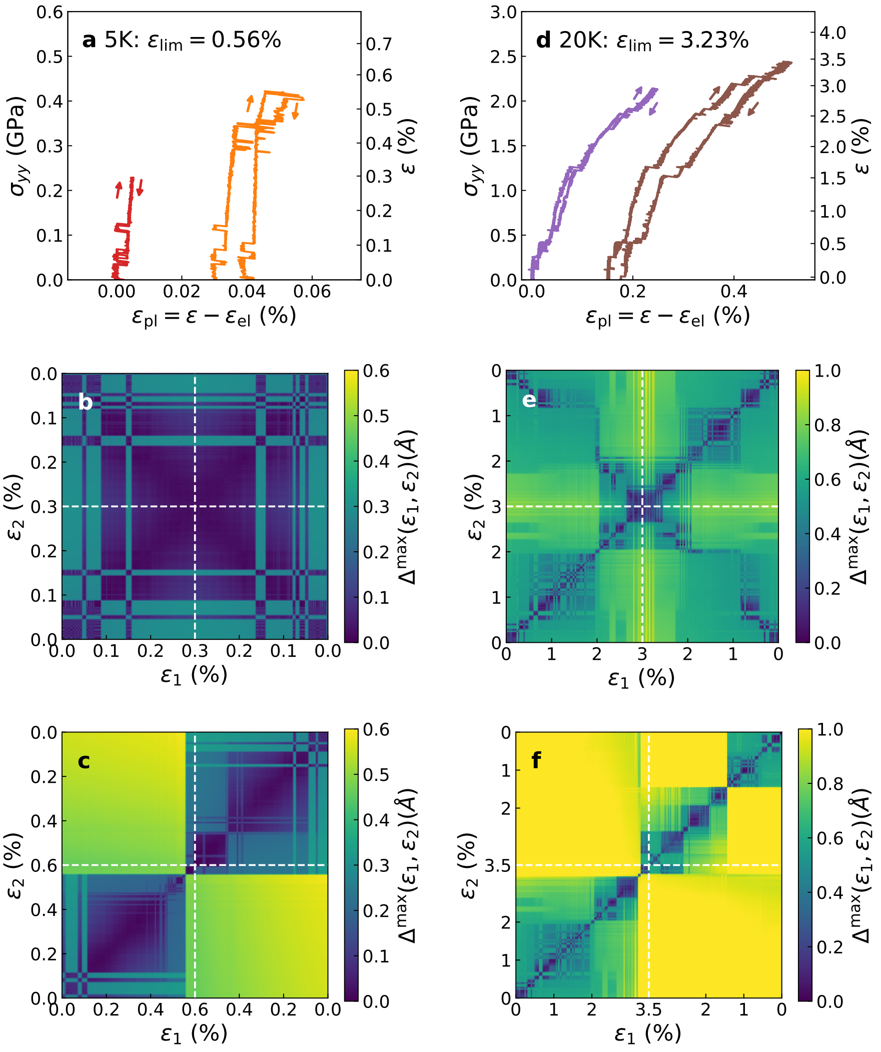

Supplementary Fig. 6 MNADM at higher temperatures. **a** Stress-strain curves of 5 K cyclic loading up to 0.3 % and 0.6 % strain. **b** MNADM for cyclic loading test up to 0.3 % strain. **c** MNADM for cyclic loading test up to 0.6 % strain. **d** Stress-strain curves of 20 K cyclic loading up to 3.0 % and 3.5 % strain. **e** MNADM for cyclic loading test up to 3.0 % strain. **f** MNADM for cyclic loading test up to 3.5 % strain.

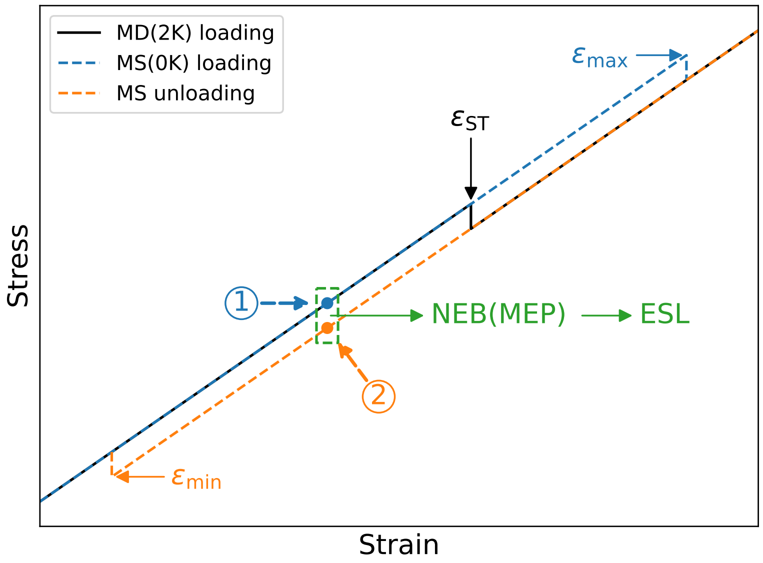

Supplementary Fig. 7 Schematic illustration of the procedure for evaluating the MEPs of an ST event at different strains. The solid black line indicates the MD simulation of the tensile loading at finite temperatures followed by energy minimization, and the ST event occurs at the strain ε_{ST}. The configurations before the ST event belong to State ①, and the configurations after the ST event belong to State ②. To obtain the entire strain range where both States ① and ② are stable, we first load the State ① configuration using molecular statics (MS, at 0 K limit) beyond ε_{ST} to ε_{max}, where the forward ST event spontaneously occurs (through a mechanical instability). Similarly, the State ② configuration is unloaded to ε_{min}, where the backward ST event occurs. The MEPs between States ① and ② are evaluated using the NEB method at different strain values within the entire strain range $(\varepsilon_{min}, \varepsilon_{max})$ to construct the ESL.

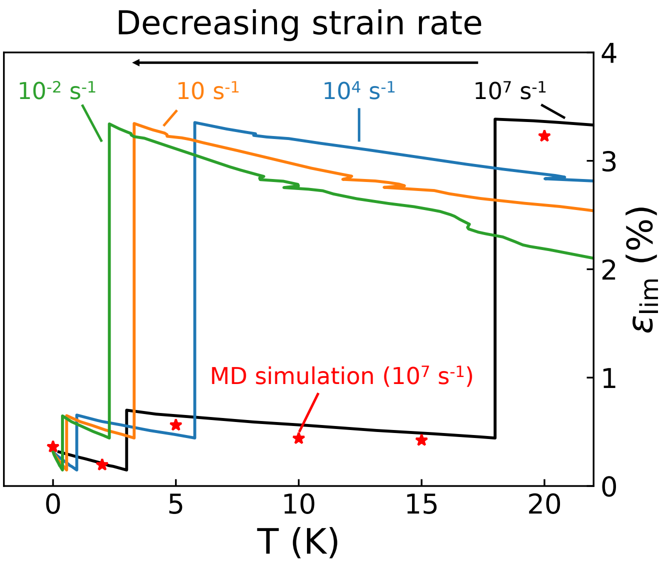

Supplementary Fig. 8 Predicted elastic limit at low strain rates. The elastic limit predicted by Supplementary Equation (3) (equation (1) in the main text) at lower strain rates.

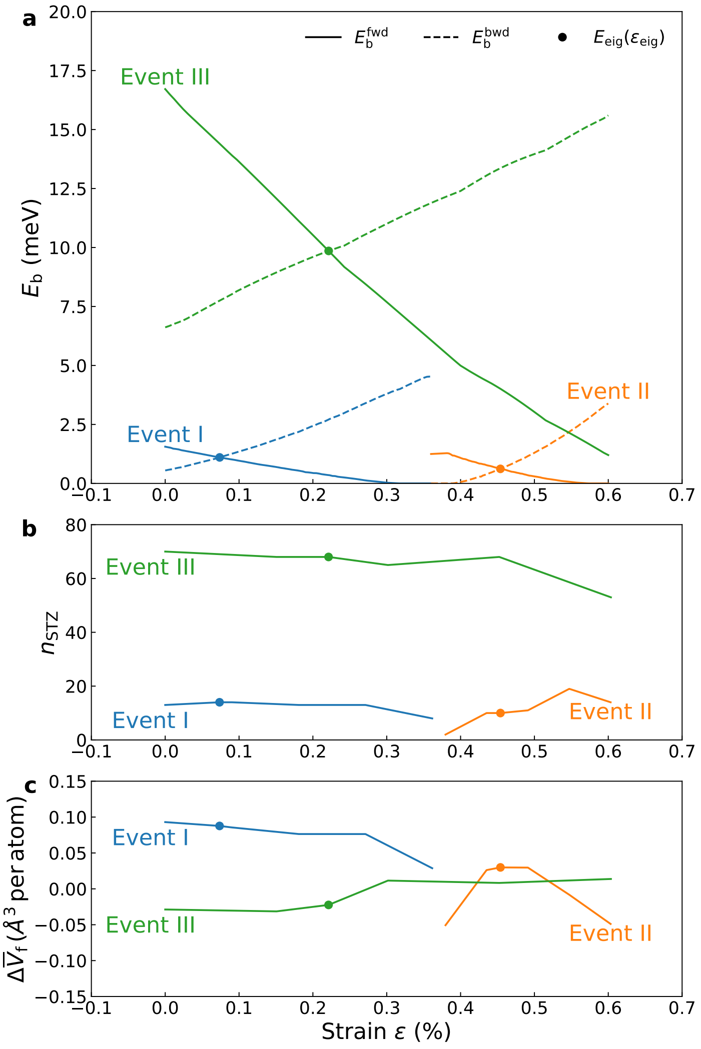

Supplementary Fig. 9 Spatial analyses of shear transformation (ST) events (I, II, III) observed at early-stage deformation. **a** Strain-dependent forward (solid lines) E_b^{fwd} and backward (dashed lines) E_b^{bwd} energy barriers. **b** Strain dependence of the shear transformation zone (STZ) size (number of atoms). **c** Strain dependence of the average free volume (FV) change per atom before and after the ST event. The values at the strain ε_{eig} corresponding to the eigen barrier E_{eig} is marked as round dots in all three plots.

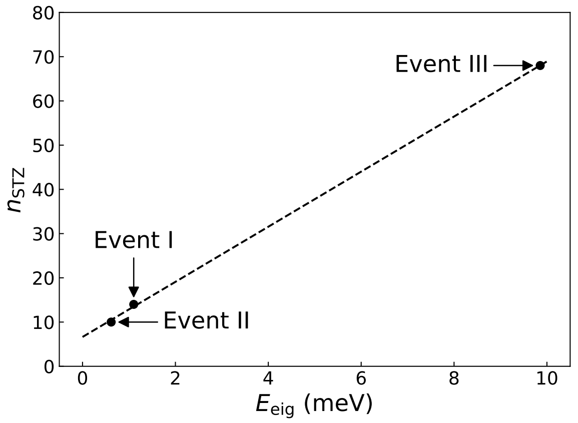

Supplementary Fig. 10 Relation between the shear transformation zone (STZ) size and the eigen barrier. STZ size (number of atoms) of shear transformation (ST) events as a function of the eigen barrier E_{eig} for the three events examined during the molecular dynamics (MD) simulations.

Supplementary References

[1] Thompson, A. P. *et al.* LAMMPS - a flexible simulation tool for particle-based materials modeling at the atomic, meso, and continuum scales. *Computer Physics Communications* **271**, 108171 (2022).

[2] Mendelev, M., Kramer, M., Becker, C. & Asta, M. Analysis of semi-empirical interatomic potentials appropriate for simulation of crystalline and liquid Al and Cu. *Philosophical Magazine* **88**, 1723–1750 (2008).

[3] Zhang, Y. *et al.* Cooling rates dependence of medium-range order development in $Cu_{64.5}Zr_{35.5}$ metallic glass. *Physical Review B* **91**, 064105 (2015).

[4] Zhang, Y., Ashcraft, R., Mendelev, M., Wang, C. Z. & Kelton, K. F. Experimental and molecular dynamics simulation study of structure of liquid and amorphous $Ni_{62}Nb_{38}$ alloy. *The Journal of Chemical Physics* **145**, 204505 (2016).

[5] Zhang, Y. *et al.* Diffusion in a Cu-Zr metallic glass studied by microsecond-scale molecular dynamics simulations. *Physical Review B* **91**, 180201 (2015).

[6] Zhang, Z., Ding, J. & Ma, E. Shear transformations in metallic glasses without excessive and predefinable defects. *Proceedings of the National Academy of Sciences* **119**, e2213941119 (2022).

[7] Zhu, T., Li, J., Samanta, A., Leach, A. & Gall, K. Temperature and strain-rate dependence of surface dislocation nucleation. *Physical Review Letters* **100**, 025502 (2008).

[8] Ryu, S., Kang, K. & Cai, W. Predicting the dislocation nucleation rate as a function of temperature and stress. *Journal of Materials Research* **26**, 2335–2354 (2011).

[9] Wang, Y. & Cai, W. Stress-dependent activation entropy in thermally activated cross-slip of dislocations. *Proceedings of the National Academy of Sciences* **120**, e2222039120 (2023).

[10] Shimizu, F., Ogata, S. & Li, J. Theory of shear banding in metallic glasses and molecular dynamics calculations. *Materials Transactions* **48**, 2923–2927 (2007).

[11] Stukowski, A. Visualization and analysis of atomistic simulation data with OVITO: the Open Visualization Tool. *Modelling and Simulation in Materials Science and Engineering* **18**, 015012 (2009).
